# Transverse and Oblique Long Bone Fracture Evaluation by Low Order Ultrasonic Guided Waves: A Simulation Study

**DOI:** 10.1155/2017/3083141

**Published:** 2017-01-15

**Authors:** Ying Li, Dan Liu, Kailiang Xu, Dean Ta, Lawrence H. Le, Weiqi Wang

**Affiliations:** ^1^Department of Electronic Engineering, Fudan University, Shanghai 200433, China; ^2^Laboratoire d'Imagerie Biomédicale, UPMC Univ Paris 06, INSERM UMR-S 1146, CNRS UMR 7371, Sorbonne Université, 75006 Paris, France; ^3^State Key Laboratory of ASIC and System, Fudan University, Shanghai 200433, China; ^4^Key Laboratory of Medical Imaging Computing and Computer Assisted Intervention (MICCAI) of Shanghai, Shanghai 200032, China; ^5^Department of Radiology and Diagnostic Imaging, University of Alberta, Edmonton, AB, Canada

## Abstract

Ultrasonic guided waves have recently been used in fracture evaluation and fracture healing monitoring. An axial transmission technique has been used to quantify the impact of the gap breakage width and fracture angle on the amplitudes of low order guided wave modes *S*0 and *A*0 under a 100 kHz narrowband excitation. In our two dimensional finite-difference time-domain (2D-FDTD) simulation, the long bones are modeled as three layers with a soft tissue overlay and marrow underlay. The simulations of the transversely and obliquely fractured long bones show that the amplitudes of both *S*0 and *A*0 decrease as the gap breakage widens. Fixing the crack width, the increase of the fracture angle relative to the cross section perpendicular to the long axis enhances the amplitude of *A*0, while the amplitude of *S*0 shows a nonmonotonic trend with the decrease of the fracture angle. The amplitude ratio between the *S*0 and *A*0 modes is used to quantitatively evaluate the fracture width and angles. The study suggests that the low order guided wave modes *S*0 and *A*0 have potentials for transverse and oblique bone fracture evaluation and fracture healing monitoring.

## 1. Introduction

Long bone fractures are typically classified by their shape complexity, locations, such as transverse, oblique, spiral, comminuted, compression, and greenstick fractures, and so forth [1]. Approximately 7.9 million patients sustain fractures in the United States annually, and up to 10% go on to have impaired bone healing, resulting in a delayed union or a nonunion [2]. More than 3 million incident fractures at a cost of $35 billion are predicted for 2025 [3]. Long bone fractures represent one of the most commonly sustained injuries following trauma and account for nearly 4% of emergency department visits in the United States each year [4]. Therefore, fracture diagnosis and subsequent healing monitoring are vital [5–7]. Although conventional X-ray radiographies are still the most common methods to evaluate fractures and monitor the subsequent healing process, ultrasonic measurement is emerging as an alternative owing to its advantages of being quick, portable, noninvasive, and inexpensive [4, 8–14]. Especially for pediatric long bone fractures, ultrasound does not necessitate exposing children to ionizing radiation, which has been linked with cancer [4, 15].

Pulse echo ultrasonic imaging showed its advantage in pediatric fracture assessment owing to children's relative thinner soft tissue compared with that of adults [10]. Li et al. proposed a split-step Fourier echo imaging method to process the signals measured by axial scanning and image the oblique cracks in the cervine long bone plate, which illustrates the potential of better resolution for ultrasound imaging [16]. However, ultrasonography still cannot detect fractures with a width less than 1 mm [9].

Axial transmission ultrasound has drawn more and more attention recently [17–20]. Two techniques have already been proposed, first arriving signal (FAS) [17, 20–23] and guided wave [19, 24–27]. 2D-FDTD simulations and in vitro experiments on the bovine tibia have been performed, and the results illustrated that both transverse and oblique cracks resulted in the amplitude loss and velocity decrease of FAS [20]. The increase of the fracture angle would impair the sensitivity of the crack width assessment. Further simulation and experimental measurements of bovine femur samples in vitro were performed [21, 28], and the influence of bone mineralization was analyzed. The results showed that the speed of the FAS could reflect the long bone fracture healing stages. An in vitro experiment on the sheep tibia confirmed that the speed of FAS had the capability to detect the long bone fracture healing stages [29]. FAS is found to be relatively low magnitude compared with the subsequently arriving guided waves and is only sensitive to changes in the periosteal regions along the propagating path of the long cortical bone [21].

Ultrasonic guided waves propagate throughout the whole waveguide with great sensitivity to the boundary conditions, such as the endosteal and periosteal regions of the long cortical bone. Previous numerical and experimental studies mainly focus on relatively high frequency [19, 26, 32]. The propagation of guided waves in long bones with different healing stages was analyzed using finite element simulation, and it was found that the cortical fracture significantly affected the propagation of guided waves [19, 26]. However, the serious mode overlap and conversion prevent accurate quantification. Many mode recognition and separation algorithms have been proposed, such as the Radon transform [33, 34], wideband dispersion reversal method [35], sparse SVD based mode extraction [36], time-frequency ridge extraction [25], and joint spectrum separation ridge extraction [37]. However, limited by the complexity of clinical measurement, the severe multimode overlap still highly complicates the ultrasonic guided wave based long cortical bone fracture evaluation.

Recently, a narrowband frequency excitation of the low order guided modes was applied to evaluate the fracture of the long cortical bone, which significantly simplifies the mode identification and signal processing [27, 38, 39]. Simulation studies and in vitro experiments demonstrated that only two fundamental modes, *S*0 and *A*0, can be excited, and the mode conversion between *S*0 and *A*0 is capable of indicating the depth of the diaphyseal cracks [27]. However, in the previous study, only transverse cracks are analyzed in the results. Actually bone fractures are often in irregular shapes and with oblique angles to the axial direction of the long bone. Aiming to investigating the impact of irregularities of cortical fractures, a 2D-FDTD simulation was carried out in this paper. We attempt to quantitatively illustrate the possibilities of using low order guided waves to evaluate the oblique fractures in long bones.

## 2. Methods

### 2.1. Guided Mode Excitation

Ultrasound propagation through a waveguide is always accompanied with the mode conversion between shear and longitudinal waves. After multiple refractions and reflections, wave packets with a similar phase will propagate together as a stable guided mode. Guided waves in the plates, also named Lamb waves, can be general grouped as symmetric and antisymmetric modes according to their different vibration features. In a plate, the vibration is constrained to the Rayleigh-Lamb equations [40]:

(1) tan⁡ph/2tan⁡qh/2+k2−q224pqk2=0symmetrictan⁡ph/2tan⁡qh/2+4pqk2k2−q22=0anti-symmetrick2=ω2Vp2,  p2=ω2VL2,  q2=ω2VT2,

where *h* is the plate thickness and the angular wave number *k* is the ratio between the phase velocity *V*_*p*_ and angular frequency *ω*. *V*_*L*_ and *V*_*T*_ are the bulk longitudinal and shear wave velocities, respectively. The numerical solutions of (1) constitute the dispersion curves which can be expressed as functions of the frequency thickness product *f* · *h*.

**Figure 1:**
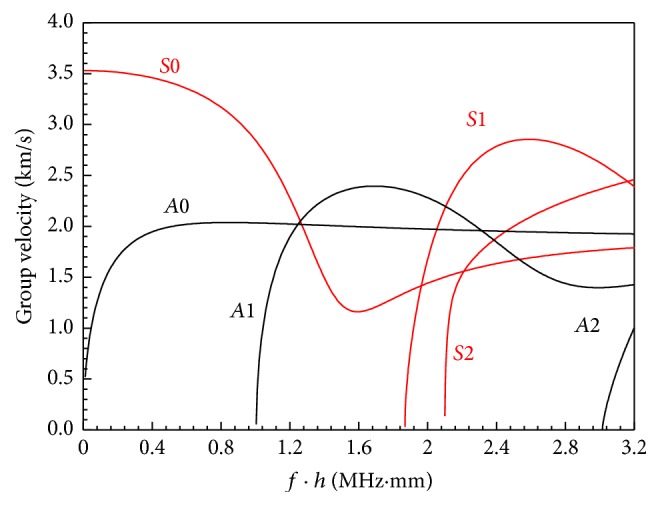
Group velocity dispersion curves of a free bovine tibia cortical bone plate. The red and black lines are the symmetric and asymmetric Lamb modes, respectively.

Group velocity dispersion curves of a free cortical bone plate are shown in Figure 1, where the red and black lines are the symmetric and antisymmetric Lamb modes, respectively. The material parameters of cortical bone are given in Table 1.

As shown in Figure 1, only two Lamb modes exist at the low frequency thickness (*f* · *h*) range (<1 MHz·mm). On the contrary, at the high *f* · *h* range (>1 MHz·mm), there exist many high order modes which brings the challenges of mode separation. Thus, simulations are performed on the low *f* · *h* range. Narrowband low frequency sinusoids (5-cycle Gaussian-modulated pulse with a 100 kHz central frequency) are employed in our simulation to merely excite two fundamental guided waves, symmetric mode *S*0 and asymmetric mode *A*0.

### 2.2. Numerical Simulation and Models

**Figure 2:**
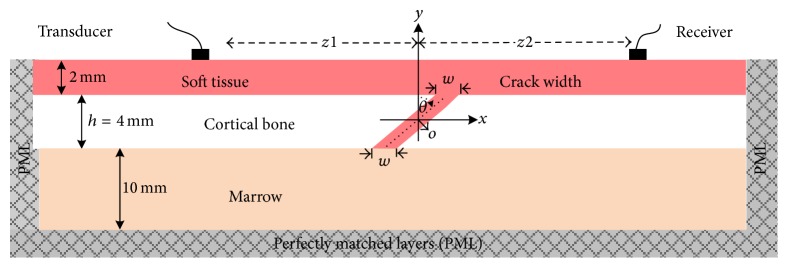
Simulation model of obliquely fractured long bone (*h* = 4 mm) with overlying 2 mm thick soft tissue and underlying 10 mm thick marrow.

The numerical simulation of the axial transmission ultrasound in the long bone is performed using a self-developed two-dimension (2D) finite-difference time-domain (FDTD) software that can numerically solve the wave field in the time and space domains [20, 23]. As shown in Figure 2, a model with dimension of 300 mm × 16 mm is used to model the obliquely fractured long bone.

The long bones are built as three layers, 2 mm thick overlaying soft tissue, 4 mm thick cortical bone, and 10 mm thick marrow. The perfectly matched layers (PML) were arranged at the two ends of the model and the beneath the marrow layer to avoid the reflection. A free boundary condition is applied on upper layer of soft tissue. In the middle of the cortical bone layer, a crack is set with a width *w* and fracture angle *θ* relative to the *y*-axis. The crack width *w* changes from 0 mm (intact) to 1 mm with an interval of 0.125 mm. The fracture angle *θ* changes from 0° (vertical) to 83°. Constrained by the model's resolution of 0.025 mm, the fracture angle cannot be set continuously. Consequently, the fracture angles are modeled with 0°, 14°, 18°, 26°, 37°, 45°, 53°, 63°, 76°, and 83°. A pair of transducers are kept in contact with the soft tissue with the incident angle of 0°. The radius of the transducer is 5 mm. The distance from the transmitter to the central *y*-axis *z*1 is 60 mm, and the distance from the receiver to the central *y*-axis *z*2 is changeable from 30 mm to 90 mm with an interval of 2.5 mm. The material properties used in the simulation are given in Table 1, and the simulation temporal discretization is 0.015 *μ*s.

### 2.3. Data Processing

In this study, a low frequency narrowband Gaussian-modulated pulse is used to avoid multimode overlapping. Thus, only two fundamental guided modes, *S*0 and *A*0, are excited in the received guide waves, so that the mode packets can be identified and separated by simple temporal windows [38, 41].

The peak amplitude of the *A*0 and *S*0 wave packets is obtained to calculate the amplitude ratio between the two modes. The energy characteristics of the *A*0 mode and *S*0 mode are investigated with fracture angles and widths variation.

The propagation delays of these two converted modes can be calculated by (2a) and (2b) [27]. If *z*1 equals *z*2, the wave packets of the two converted modes merge into a mixed wave packet propagating between the original *S*0 and *A*0 modes

(2a) TA0S0=z1VS0+z2VA0,

(2b) TS0A0=z1VA0+z2VS0.

At 100 kHz, the duration of the 5 cycle excitation Gaussian-modulated pulse is 50 *μ*s, and the group velocities of *S*0 and *A*0 are 3.95 km/s and 1.51 km/s, respectively. We measure the peaks of the two wave packets in time domain, which are the maximum amplitudes of the two original modes *S*0 and *A*0 and calculate the amplitude ratio between them for use in evaluating the long bone crack width with different fracture angles.

## 3. Results

**Figure 3:**
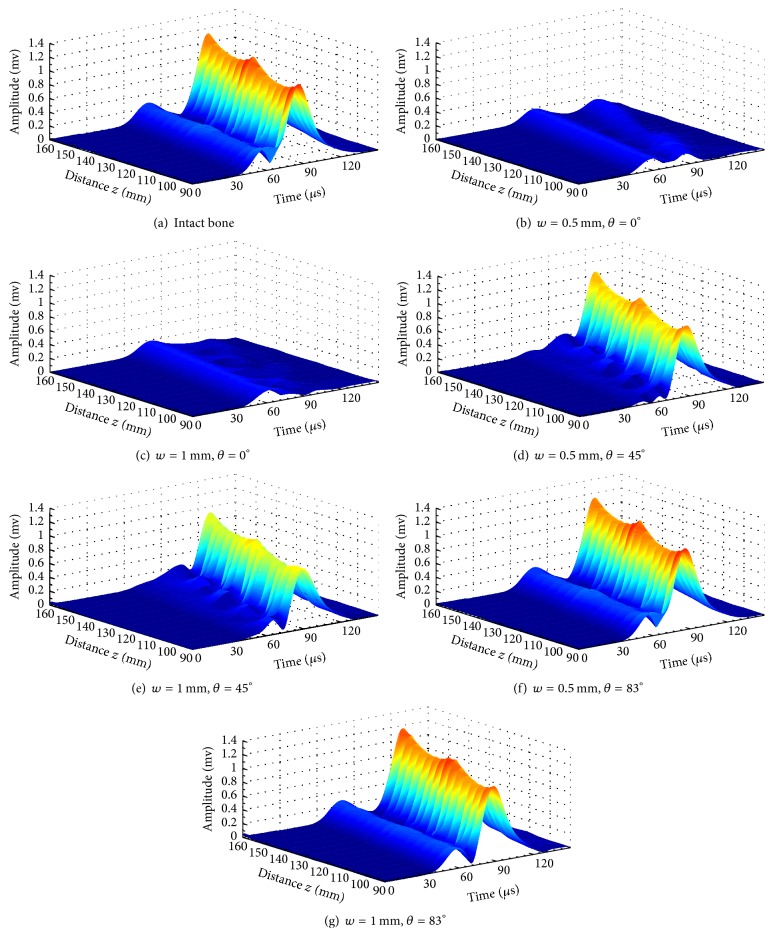
The envelope diagrams of the received signals (90 mm < *z* < 150 mm) with different fracture degree and oblique angles, (a) intact model, (b) transverse fracture model with 0.5 mm wide crack, (c) transverse fracture model with 1.0 mm wide crack, (d) 45° oblique fracture model with 0.5 mm wide crack, (e) 45° oblique fracture model with 1.0 mm wide crack, (f) 83° oblique fractured model with 0.5 mm wide crack, and (g) 83° oblique fracture model with 1.0 mm wide crack.

Figure 3 shows the envelope curves extracted from fractured long bones with different fracture degrees and fracture oblique angles. The envelope amplitude is depicted in different colors with maxima in red and 0 in gray. The propagation distance *z* varies from 90 mm to 150 mm with an interval of 2.5 mm.

Figure 3(a) shows the envelopes of the received waveforms with two Lamb modes *S*0 and *A*0 in the intact bone. The amplitude of antisymmetric mode *A*0 is much higher than that of *S*0, because of the perpendicular incidence and reception angles. No converted modes are observed in this model. However, in Figures 3(b) and 3(c) for transverse fracture with 0.5 mm and 1 mm wide cracks, both the *S*0 and *A*0 amplitudes attenuate significantly. As shown in Figures 3(d) and 3(e), the converted modes can be observed from the results of the 45° oblique fracture. According to the velocity analysis, the converted modes usually exist between the two original modes *S*0 and *A*0. Furthermore, the peak amplitudes of the conversion modes and original mode are difficult to be extracted due to mode overlapping. For larger fracture angle models in Figures 3(f) and 3(g), it seems that the original mode energy still can transmit through the cracks without obvious appearance of the converted modes. With a fixed fracture angle, the increasing of the crack width may lead to the amplitude reduction of both *S*0 and *A*0, such as in Figures 3(a), 3(b), and 3(c). In Figures 3(b), 3(d), and 3(f), with a fixed crack width, the increasing fracture angle may lead to the amplitude increasing of transmitted energy of the *A*0 mode, but the change of the *S*0 amplitude is nonmonotonic. To quantitatively illustrate the mode conversion, we further investigate different models with crack and fracture angle variation.

### 3.1. *A*0 Mode Amplitude Analysis

**Figure 4:**
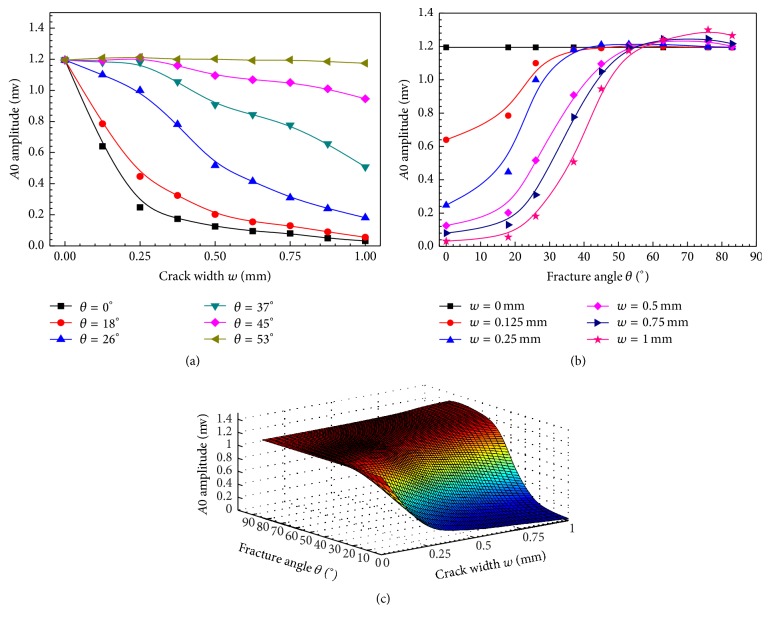
Amplitudes of *A*0 change as functions of the crack width *w* and the fracture angle *θ*; (a) with a fixed *θ*, the *A*0 amplitude curves with *w* variation; (b) with a fixed *w*, the *A*0 amplitude curves with *θ* variation; and (c) the B-spline fitting results of *A*0 amplitude to *θ* and *w*.

Figure 4(a) presents the *A*0 amplitude changes as functions of the crack width and fracture angles. The propagation distance *z* is fixed at 120 mm. With the crack width increasing, a decrease trend of the *A*0 amplitude can be observed, which is sensitive to the small crack (*w* < 0.25 mm).

The *A*0 amplitudes decrease more than 95% in the 0° and 18° fractures, but they are almost constant as *w* increases to large angles (*θ* > 53°).  Figure 4(b) shows the variation of the *A*0 amplitude with the fracture angle. The *A*0 amplitude obtained from the intact model is set as a benchmark. The *A*0 amplitude shows three stages in the increase of the fracture angle: a slow increase for small angles (*θ* < 20°), a rapid increase for the range of 20°*～*50°, and a final slow increase for large angles (*θ* > 50°).  Figure 4(c) shows the *A*0 amplitude changes with the crack width and fracture angle in three dimensions. A B-spline fitting is performed to obtain the smooth amplitude functions. It shows that, comparing with the wide cracks and transverse fractures, the narrow cracks or the large angle oblique cracks can enhance the *A*0 mode transmission with larger *A*0 amplitude. The sensitivity of the *A*0 amplitude to the crack width and fracture angle shows potentials for fracture evaluation.

### 3.2. *S*0 Mode Amplitude Analysis

**Figure 5:**
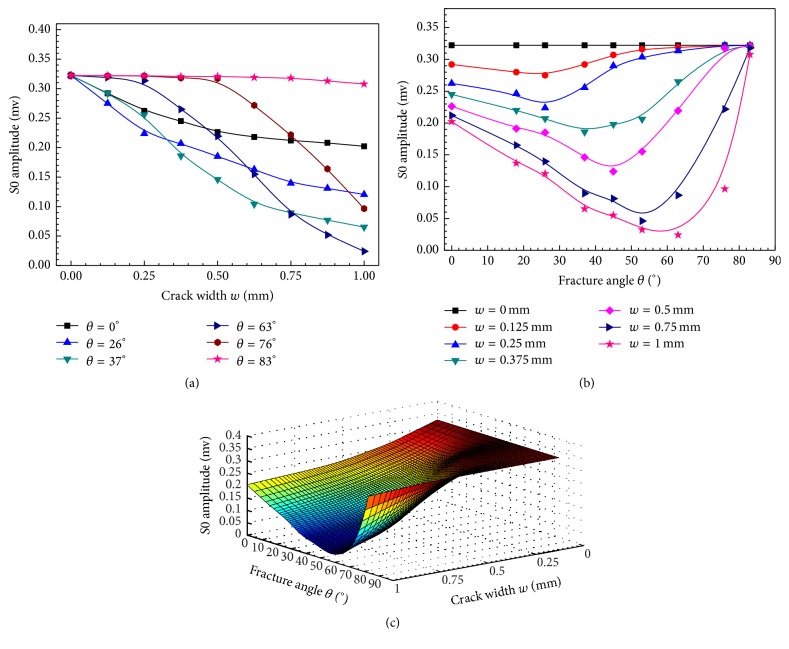
Amplitudes of *S*0 change as functions of crack width *w* and fracture angle *θ*; (a) with a fixed *θ*, the *S*0 amplitude curves with *w* variation; (b) with a fixed *w*, the *S*0 amplitudes curves with *θ* variation; and (c) the B-spline fitting results of *S*0 amplitude to *θ* and *w*.

Figure 5(a) presents the *S*0 amplitude changes as functions of the crack width with different fracture angles. The propagation distance *z* is fixed at 120 mm. The *S*0 amplitude obtained in an intact model is used as a benchmark. With a fixed fracture angle, the *S*0 amplitude decreases with the crack widening. As shown in Figure 5(b), with a fixed crack width, the *S*0 amplitude shows a nonmonotonic trend with the fracture angle variation, and the minimal *S*0 amplitude is obtained at the range of 30° to 60°, which is smaller than the amplitude obtained in the model with a transverse fracture. The *S*0 amplitude decreases as the fracture angle increases, and then it increases as the fracture angle further increases, ultimately coming close to the value of the intact model at 83°.  Figure 5(c) shows the B-spline fitting results of the *S*0 amplitude variation with the crack width and fracture angle in three-dimensional form. It can be found that the *S*0 amplitude generally decreases as the crack width increases, but it also shows a nonmonotonic trend to the fracture angle.

### 3.3. Impact of Crack Width and Fracture Angle on *S*0/*A*0

The above results indicate that the *S*0 and *A*0 amplitudes can reflect the *w* change of fractured long bones with different angles. However, in actual clinical use, the magnitudes of the guided modes are easily affected by many factors such as the coupling conditions and excitation energy. Thus, the amplitude ratio between *S*0 and *A*0 can be adopted as a more robust parameter.

**Figure 6:**
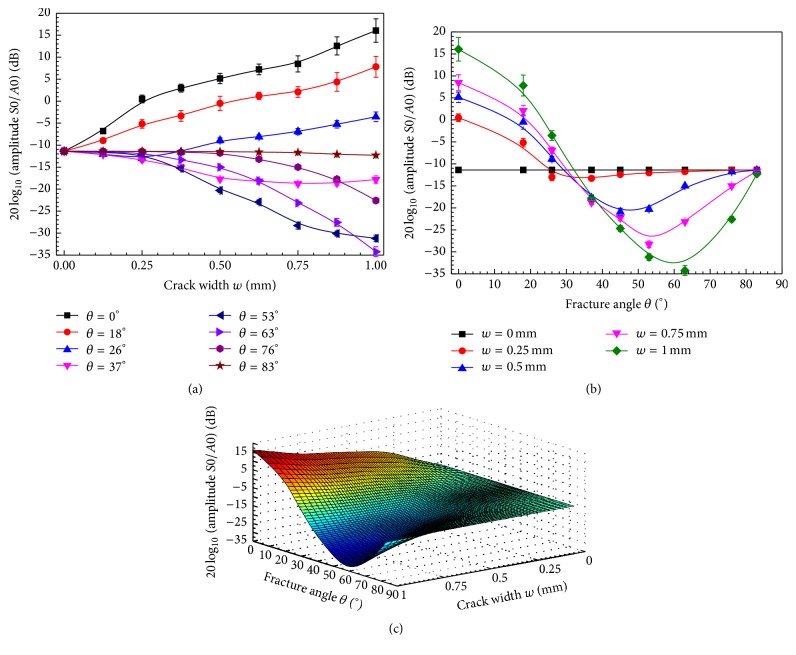
Amplitude ratios (dB) between *S*0 and *A*0 varying as functions of crack width *w* and fracture angle *θ*; (a) with a fixed *θ*, the amplitude ratio (dB) changes with *w*, where the error bars denote the standard errors for results obtained at different propagation distances *z* (90 < *z* < 150 mm); (b) with a fixed crack width *w*, the amplitude ratio (dB) changes with *θ*, where the error bars denote the standard errors for results obtained at different propagation distances *z* (90 < *z* < 150 mm); and (c) distance *z* = 120 mm, the B-spline fitting results of the amplitude ratios (dB) varying with fracture angle *θ* and crack width *w*.

To investigate the impact of the propagation distance, the average amplitude ratios are computed from the multichannel signals (90 < *z* < 150 mm with an interval of 2.5 mm). Figures 6(a) and 6(b) present the amplitude ratio changes with the variance of *w* and *θ*, respectively, where the error bar denotes the root mean square errors (RMSE) of the ratios obtained at different propagation distances for each fractured model.

As shown in Figure 6(a), for small angles (*θ* < 26°), the amplitude ratio increases with *w*. Comparing with the intact model, the amplitude ratios between the *S*0 and *A*0 modes in the fracture bone increase by values of 27.4 dB and 7.8 dB for 0° and 26°, respectively. However, for large angles (*θ* > 26°), the amplitude ratio decreases with the crack width by an average of 12.4 dB. Comparing to the intact model, with the oblique fracture angles of 53° and 63°, amplitude ratios between the *S*0 and *A*0 modes decrease by 19.8 dB and 22.9 dB, respectively.

Figure 6(b) presents the amplitude ratio of *S*0 and *A*0 varying with the fracture angle. It can be seen that the amplitude ratio curves first decrease as the fracture angle increases and then increase with angle increases. For 0.25 mm and 1 mm wide cracks, the turning points are approximately 35° and 60°. It can be found that, with a wide crack, the turn points of the amplitude ratio curves appear out with large angle.

The results reveal that the propagation distance has a small impact on the amplitude ratio with an average RSME of 0.78 dB. Furthermore, it is notable in Figures 6(a) and 6(b) that the largest RMSE of the amplitude ratio between the *S*0 and *A*0 modes can be obtained in the fractured bones with a small oblique angles (0°, 26°, and 37°) and large crack widths (*w* > 0.5 mm). For other fracture angles (*θ* > 26°), the average RSME of the amplitude ratio between the *S*0 and *A*0 modes is 0.46 dB. The small RSME reveals that the propagation distance variation between 60 mm and 150 mm has a small impact on the amplitude ratio parameter.

Figure 6(c) is the B-spline fitting result of the amplitude ratio parameter varying with the fracture angle *θ* and crack width *w* at a 120 mm distance. As shown in Figure 5(a), the line cross actually indicates that, in the oblique fractured bone model, with the crack widening the decline slopes of the *S*0 amplitude curves are different. The decline slope corresponding to the 63° fracture is more negative than those of other angles. Such a phenomenon also can be learned from the amplitude ratios curves between the *S*0 and *A*0 modes in Figure 6(c). It can be seen that the amplitude ratio may be able to indicate the oblique angle and the crack width of the bone fractures.

## 4. Discussion

This study presents a quantitative investigation of using low order guided wave modes to evaluate long bone fractures with oblique fracture angles. A 2D-FDTD simulation is performed in a three-layer model. Using a 100 kHz narrowband excitation, only two guided wave modes, *S*0 and *A*0, are excited. The impact of the crack width and fracture angle on the *S*0 and *A*0 amplitudes is thoroughly studied. The crack width increase leads to amplitude decreases for both the *A*0 and *S*0 modes (Figures 4(a) and 5(a)). The *A*0 amplitude shows a monotonic relationship with the fracture angle (Figure 4(b)), while the *S*0 amplitude shows a nonmonotonic relationship (Figure 5(b)). Although only simulation is performed in this study, the simulation results yielded interesting findings, including the use of the amplitude ratio to evaluate the crack width and fracture angle (Figures 6(a) and 6(b)). These findings illustrate the potential of guided mode conversion for the quantitative prediction of the cortical bone fracture degree and healing status.

To reduce the influence of the coupling and excitation, the amplitude ratio between *S*0 and *A*0 is adopted to evaluate the long bone fracture degree. The amplitude ratio shows a two-stage change with the fracture angle (Figure 6(b)). In the first stage, the amplitude ratio decreases as the angle increases, and the average decrease at different widths is 6.1 dB/10°. In the second stage, the amplitude ratio increases with the further increase in the angle, and the average increase at different widths is 4.9 dB/10°. The increase of crack width raises the amplitude ratio for small angles (*θ* < 26°) (Figure 6(a)), while it decreases the amplitude ratio for large angles (*θ* > 26°). The amplitude ratio between *S*0 and *A*0 shows a great capacity for fracture width detection with different fracture angles, even for very small crack widths (*w* < 1 mm): 27.4 dB/mm for a transverse fracture, 19.2 dB/mm for a 18° oblique fracture, −19.8 dB/mm for a 53° oblique fracture, and −22.9 dB/mm for a 63° oblique fracture (Figure 6(a)).

It has been demonstrated that when the cortical bone thickness (*h*) is much larger than the longitudinal wave length (*λ*), the speed of FAS is larger than that of the *S*0 mode, but for very thin plate, the speed of the FAS signal approaches the velocity of *S*0 mode [17]. Numerical and experimental results showed that the detection accuracy of the amplitude of FAS for the transverse and oblique crack widths was approximately 2 dB/mm in a 6 mm thick long bone plate at an excitation of 200 kHz (*h* ≈ 0.3*λ*) [20]. In the performed study, as the excitation frequency is 100 kHz and the cortical bone plate thickness is 4 mm (*λ* ≈ 40 mm, *h* ≈ 0.1*λ*), the FAS can be regarded as the ultrasonic Lamb mode *S*0. Compared with high frequency excitation, these simulation results show that the amplitude ratio between *S*0 and *A*0 may also be used to detect the crack width. In addition, for the oblique fracture model with a 37~63° fracture, the *S*0 amplitude significantly decreases as the crack width increases, so it is relatively hard to measure mode *S*0. Therefore, the incident angle should be optimized to increase the excitation of *S*0. The different distance signal results reveal that the propagation distance has a small impact on the amplitude ratio, with an average RSME of 0.78 dB. These results indicate the theoretical feasibility for the clinical application.

In the performed simulation, we consider that the crack surface is uniform, but it is actually irregular, which will affect the propagations of *S*0 and *A*0. Moreover, the crack region is filled with soft tissue in our simulation, while actually the tissue in the crack site will gradually recover, with a callus appearing and changing during the healing [26, 42–44]. Further experiments are needed to validate our hypothesis that the amplitude ratio between the *S*0 and *A*0 modes can indeed facilitate long bone fracture and healing process evaluation.

## 5. Conclusions

The impacts of the crack width and fracture angle on the low order guided wave *S*0 and *A*0 amplitudes were studied in a three-layer model (soft tissue, cortical bone, and marrow) using the 2D-FDTD simulation. The results show that the *S*0 and *A*0 amplitudes decrease as the crack width increases. The *A*0 amplitude increases as the fracture angle increases, while the *S*0 amplitude firstly decreases and then increases. To avoid the influence of coupling conditions and excitation signals, the use of the amplitude ratio between *S*0 and *A*0 is proposed to evaluate crack width changes at different fracture angles. The results indicate that the amplitude ratio between *S*0 and *A*0 is sensitive to the crack width in fractures with different oblique angles. The amplitude ratio shows good capability for crack width evaluation, with sensitivities of 25.1 dB/mm for a transverse fracture, 18.7dB/mm for an 18° oblique fracture, and −23.3 dB/mm for 53° oblique fracture. The average RSME of 0.78 dB for different propagation distances indicates the small distance impact on the amplitude ratio. Thus, the amplitude ratio between *S*0 and *A*0 has the capability of reflecting the long bone fracture status, including the crack width and angle. The variation of the amplitude ratio with the crack width and fracture angle was further discussed, which shows its good potential for monitoring the fracture angle and crack width in fractured long cortical bone.

## Figures and Tables

**Table 1:** Parameters of cortical bone and soft tissue.

Tissue	*ρ* (g/m^3^)	*V*_*L*_ (km/s)	*V*_*T*_ (km/s)	Attenuation (dB·cm^−1^·MHz^−1^)
Cortical bone [20]	2.00	4.20	2.00	5.09
Soft tissue [30]	0.92	1.47	0.10	1.20
Marrow [31]	0.10	1.40	0.07	0.80
